# Increased clonality among *Neisseria gonorrhoeae* isolates during the COVID-19 pandemic in Amsterdam, the Netherlands

**DOI:** 10.1099/mgen.0.000975

**Published:** 2023-04-06

**Authors:** H.C.A. Zondag, J. de Korne-Elenbaas, S.M. Bruisten, H.J.C. de Vries, A.P. van Dam

**Affiliations:** ^1^​ Department of Infectious Diseases, Public Health Laboratory, Public Health Service of Amsterdam, Amsterdam, Netherlands; ^2^​ Department of Medical Microbiology, Amsterdam UMC, University of Amsterdam, Amsterdam Institute for Infection & Immunity (AI&II), location Amsterdam Medical Centre, Amsterdam, Netherlands; ^3^​ Department of Dermatology, Amsterdam UMC, University of Amsterdam, Amsterdam Institute for Infection & Immunity (AI&II), location Amsterdam Medical Centre, Amsterdam, Netherlands; ^4^​ Department of Infectious Diseases, Centre for Sexual Health, Public Health Service Amsterdam, Amsterdam, Netherlands

**Keywords:** *Neisseria gonorrhoeae*, sexually transmitted diseases, COVID-19 pandemic, lockdown, genomics

## Abstract

Distancing measures during the COVID-19 lockdown led to a temporary decrease of casual sex partners among clients of the Centre for Sexual Health (CSH) in Amsterdam, the Netherlands. We investigated the effect of this change on the genotypic and phenotypic distribution of *Neisseria gonorrhoeae* (*Ng*) isolates from CSH patients. From each *Ng-*positive patient we sequenced one isolate, resulting in 322 isolates which constituted two groups: 181 isolates cultured from 15 January to 29 February 2020 (before the first lockdown) and 141 cultured from 15 May to 30 June 2020 (during the first lockdown). Patient characteristics showed significantly more symptomatic patients and significantly fewer reported sex partners during the lockdown. Phenotypic data showed an increase in low-level azithromycin resistance and ceftriaxone susceptibility during the lockdown, and this remained after the study period. The diversity in sequence types (STs) decreased slightly during the lockdown. A shift occurred from ST 8156 being predominant before lockdown to ST 9362 during lockdown and a remarkably low median SNP distance of 17 SNPs was found between ST 9362 isolates obtained during lockdown. These findings reflect restricted travel and the change in sexual behaviour of CSH clients during the lockdown, with a potentially increased local transmission of the ST 9362 strain during this period, which led to genotypic and phenotypic changes in the *Ng* population. This shows that public health measures have far-reaching consequences and should be considered in the surveillance of other infectious diseases.

## Data Summary

Raw sequence data are available in the European Nucleotide Archive under project number PRJEB55899 and individual accession numbers can be found in Table S1 (available in the online version of this article). Assemblies are available in the PubMLST *Neisseria* database (https://pubmlst.org/organisms/neisseria-spp). PubMLST identifiers and additional metadata can also be found in Table S1. The bioinformatic pipeline used in this study can be found on Github (https://github.com/jolindadekorne/Diversity-In-Neisseria-Gonorrhoeae-during-Lockdown).

**Impact Statement**

*Neisseria gonorrhoeae (Ng*) is a worldwide prevalent sexually transmitted infection. The molecular epidemiology of this bacterium is closely monitored, providing information on circulation genotypes associated with antimicrobial resistance over time. However, major events such as the COVID-19 pandemic causes behavioural changes in patient populations and impact infectious diseases. By analysing patients who visited the Centre of Sexual Health in Amsterdam before (January–February) and during (May–June) the first lockdown in 2020 in the Netherlands, we found a shift in the genotypic and phenotypic distribution of *Ng* and the emergence of a highly clonal *Ng* strain, suggesting local transmission. This supports previous research that found reduced numbers of sex with casual partners among this population during the lockdown. This work shows that public health measures have an impact on the epidemiology of other infectious diseases, which should be taken into consideration for public health surveillance.

## Introduction

The COVID-19 pandemic has had an extraordinary global impact on public health [1]. Also, the epidemiology of sexually transmitted infections (STIs) has been impacted by the COVID-19 pandemic [2]. Gonorrhoea, caused by the sexually transmitted pathogen *Neisseria gonorrhoeae* (*Ng*)*,* is one of the most prevalent bacterial STIs worldwide and its prevalence is globally on the rise [3]. This rising trend has also been reported in Europe, including the Netherlands, where before the pandemic the prevalence of gonorrhoea and other bacterial STIs increased, especially among men who have sex with men (MSM) [4–6]. However, interpreting the number of STI cases during the COVID-19 pandemic has been challenging, since social measures and large scale COVID-19 testing temporarily restricted STI testing at Centres for Sexual Health (CSH) [4, 7].

In the Netherlands, efforts to reduce and contain the transmission of severe acute respiratory syndrome coronavirus 2 (SARS-CoV-2) started with gradual measures since the first notified infection on 27 February 2020. The first lockdown with additional measures was put into place on 16 March 2020, from then all events were cancelled, gatherings of three and more persons were discouraged, and all shops (except grocery shops), schools and leisure areas were closed (Table S2). In general, 1.5 metres of physical distance was enforced both inside and outside, and no more than three adult visitors were allowed in a household per day. International travel was restricted to essential travel only and, nationally, people were encouraged to stay at home. From mid-May onwards, schools, shops, museums, bars and restaurants were gradually reopened with restrictions. However, during this time, any form of travel and especially international travel remained highly reduced [8]. The healthcare at the CSH in Amsterdam was also impacted by the restrictions of the lockdown (Table S2). Between 23 March and June first routine human immunodeficiency virus (HIV)/STI testing was halted for asymptomatic clients unless urgent PrEP prescription was needed [9].

The perceived risk of SARS-CoV-2 infection and public health measures were found to be associated with a change in sexual behaviour [10, 11]. A recent study from Bilsen *et al*. [9] found that distancing measures during lockdown led to a temporary decrease in casual sex partners and a relatively low STI positivity rate among clients of the CSH in Amsterdam [9]. Besides the influence of this behavioural change on the number of STI cases, it could also have had an influence on STI epidemiology due to changes in transmission networks. In this study, we aimed to investigate the influence of the change in sexual behaviour of CSH clients during the first lockdown in Amsterdam on the genotypic and phenotypic distribution of *Ng* isolates from CSH patients.

## Methods

### Isolate selection

Two study periods of equal duration were selected before and during the lockdown based on similarity of patient groups regarding sex and sexual orientation. The study periods were also chosen based on the number of *Ng* isolates obtained during each period, because during the first 8 weeks of the lockdown, access to the CSH was highly restricted, which subsequently strongly reduced the number of gonorrhoea cases. *Ng* isolates from all culture-positive patients who visited the CSH between 15 January and 29 February and between 15 May and 30 June in 2020 were included. The period 15 January to 29 February 2020 will be referred to as ‘before lockdown’ and the period 15 May to 30 June 2020 will be referred to as ‘during lockdown’ throughout this study. According to our routine clinical practice, *Ng-*positive patients always received treatment with a single intramuscular dose of 1 g ceftriaxone, irrespective of having symptoms. Therefore, patients who were *Ng-*positive both before and during lockdown were included in both sets of patient groups, since these can be considered as two separate infection episodes. From each *Ng-*positive patient, one isolate was selected with prioritization of the rectal isolate in case multiple isolates were obtained from a single patient. To prevent SARS-CoV-2 transmission at the CSH, the sampling policy no longer included pharyngeal sampling during the lockdown. Therefore, pharyngeal *Ng* isolates obtained before the lockdown were not included in this study.

### Phenotypic analysis

At the Public Health Laboratory, minimum inhibitory concentrations (MICs) for azithromycin and ceftriaxone are routinely determined for all *Ng* isolates obtained from CSH patients, using E-tests according to the manufacturer’s instruction (bioMérieux). The clinical breakpoint used in this study for ceftriaxone resistance was MIC >0.125 mg l^−1^ and for azithromycin MIC ≥1 mg l^−1^ (epidemiological cut-off value), according to EUCAST guidelines v12.

To determine whether phenotypic trends observed during the study period were already seen before the study period or still present in the months afterwards, azithromycin and ceftriaxone MIC distributions of anogenital isolates obtained 2.5 months before (15 August – 30 September 2019) and 2.5 months after the study period (15 September – 1 November 2020) were compared to MICs of isolates obtained during the study period. The interval of 2.5 months was chosen because the study periods before and during lockdown also had an interval of 2.5 months. Pharyngeal isolates were excluded from the phenotypic data because pharyngeal isolates were not included during the study period.

### Isolate preparation and whole genome sequencing

All selected samples were taken from the −80 °C freezer and grown overnight on chocolate blood agar plates. DNA was extracted from pure cultures in DNA/RNA shield buffer using the ZymoBIOMICS Magbead DNA kit (Zymo Research). The Nextera XT DNA Library Preparation kit with Integrated DNA Technologies for Illumina DNA/RNA Unique Dual Indexes (Illumina) was used for library preparation. Whole genome sequencing (WGS) was performed by 150 bp paired-end sequencing using an Illumina NovaSeq 6000 at the sequencing facility BaseClear B.V. (L457; NEN-EN-ISO/IEC 17025).

### Quality control and assembly

Raw reads were filtered and trimmed using fastp v0.20.1 [12]. Mean coverage depth and the percentage of covered reference genome bases were calculated with the SAMtools package v1.15 by first mapping the reads to the reference genome FA1090 (NC_002946.1) with BWA-MEM2 v2.1 [13]. Genomes were assembled using the ‘isolate’ option in SPAdes v.3.15.3 [14]. QUAST v5.0.2 was used to assess the quality of the assembly and to identify assemblies with aberrant assembly lengths or GC content, which could be indications of read contamination [15]. For these isolates, reads not belonging to *Ng* (taxid:485) were identified and filtered out with Kraken2 v2.1.1 [16]. Filtered reads were assembled again and quality was again assessed.

### Typing and determination of SNP distances

All assemblies were annotated automatically by uploading them to the PubMLST *Neisseria* database. Multi-locus sequence types (MLSTs) were extracted and novel MLSTs were assigned novel STs [17]. Snippy v.4.6.0 (https://github.com/tseemann/snippy) was used for variant calling, by first mapping reads to the reference genome FA1090 followed by identifying SNPs between the isolate and reference genome. Only SNPs with a minimum base quality of 13, a minimum read coverage of 10× and a read concordance of 90 % were reported (default settings). The ‘core’ option of Snippy was used to create a core genome alignment of all isolates. To determine genetic diversity between isolates of the *Ng* population before or during the lockdown with maximimum resolution, SNP distances were determined in a pairwise manner between all possible combinations of isolates with identical STs without prior recombination filtering using snp-dists v.0.7.0 (https://github.com/tseemann/snp-dists). In addition, recombination filtering was applied and a recombination filtered core SNP tree was created using Gubbins v2.4.1. Isolate pairs that differed by <10 recombination filtered SNPs were identified, as this is the threshold previously defined for identical isolates that are putatively transmitted between individuals [18, 19]. Transmission clusters were defined as networks of isolates that differ by <10 recombination filtered SNPs. The bioinformatic pipeline was managed using Snakemake v7.2.1 [20].

### Statistical analyses

Sociodemographic and clinical data were extracted from electronic patient files. Chi square or Fisher’s exact tests were used to compare participant characteristics from before and during lockdown and to analyse its associations with the most prevalent ST during the lockdown. A *P*-value of ≤0.05 was deemed significant. Diversity in MLSTs was assessed by calculating the Simpson’s diversity index, using the R package abdiv. SNP distances between groups were compared with the Wilcoxon rank test, for which *P*≤0.001 was deemed significant. Data were analysed using RStudio (version 1.2.5033).

### Ethics statement

All clients of the CSH in Amsterdam were informed of the ‘opt-out’ system regarding research on remnants of patient material. All data were pseudonymized before analysis.

## Results

### Patient characteristics

A total of 322 *Ng* isolates were included in the study, consisting of 181 isolates before lockdown and 141 during lockdown. Demographic characteristics of the patient groups before (181 patients) and during (141 patients) lockdown were comparable regarding sex and sexual orientation, as expected because study periods were chosen based on similarity of these characteristics. Five patients were *Ng-*positive both before and during lockdown and therefore included in both groups. The patient groups differed significantly in number of sex partners (*P*=0.009) and symptomatology (*P*=0.006) (Table 1). During the lockdown, patients reported fewer sex partners in the past 6 months and a higher proportion of patients were symptomatic, compared to before the lockdown.

**Table 1.** Clinical and demographic data of patients before (15 January – 29 February) and during (15 May – 30 June) the lockdown in 2020

	Before lockdown, *N*=181 (%)	During lockdown, *N*=141 (%)	OR/*P*-value
**Sex**			0.966
Male	171 (94)	133 (94)	
Female	10 (6)	8 (6)	
**Age, years**			0.421
<25	34 (19)	36 (26)	
25–34	79 (44)	62 (44)	
35–44	47 (26)	30 (21)	
≥45	21 (12)	13 (9)	
**Anatomical location**			0.850*
Cervix	3 (2)	3 (2)	
Anus	117 (65)	87 (62)	
Urethra	54 (30)	47 (33)	
Vagina	7 (4)	4 (3)	
**Country of origin**			0.697*
The Netherlands	93 (51)	65 (46)	
Suriname+Dutch Antilles	17 (9)	19 (13)	
Europe+Turkey	28 (15)	28 (20)	
Middle+South America	20 (11)	11 (8)	
Asia	14 (8)	10 (7)	
Africa	7 (4)	5 (4)	
Other	2 (1)	2 (1)	
Unknown	0 (0)	2 (1)	
**Sexual orientation**			0.707*
MSM	158 (86)	122 (87)	
MSW	11 (6)	11 (8)	
Female	10 (6)	8 (6)	
Transgender	2 (1)	0 (0)	
**Sex work**			
Yes	15 (8)	11 (8)	0.859
No	166 (92)	130 (92)	
**Number of sex partners in last 6** **months**			0.009†
0–1	6 (3)	10 (7)	
2–4	38 (21)	47 (33)	
5–9	46 (25)	37 (26)	
10–19	32 (18)	24 (17)	
20–49	43 (24)	16 (11)	
≥50	15 (8)	7 (5)	
**Symptomaticity**			0.006†
Symptomatic	48 (27)	58 (41)	
Asymptomatic	133 (73)	84 (59)	

*Fisher’s exact test.

† *P*≤0.05.

MSM, men who have sex with men; MSW, men who have sex with women.

### Distribution of STs changed during lockdown

**Fig. 1.**
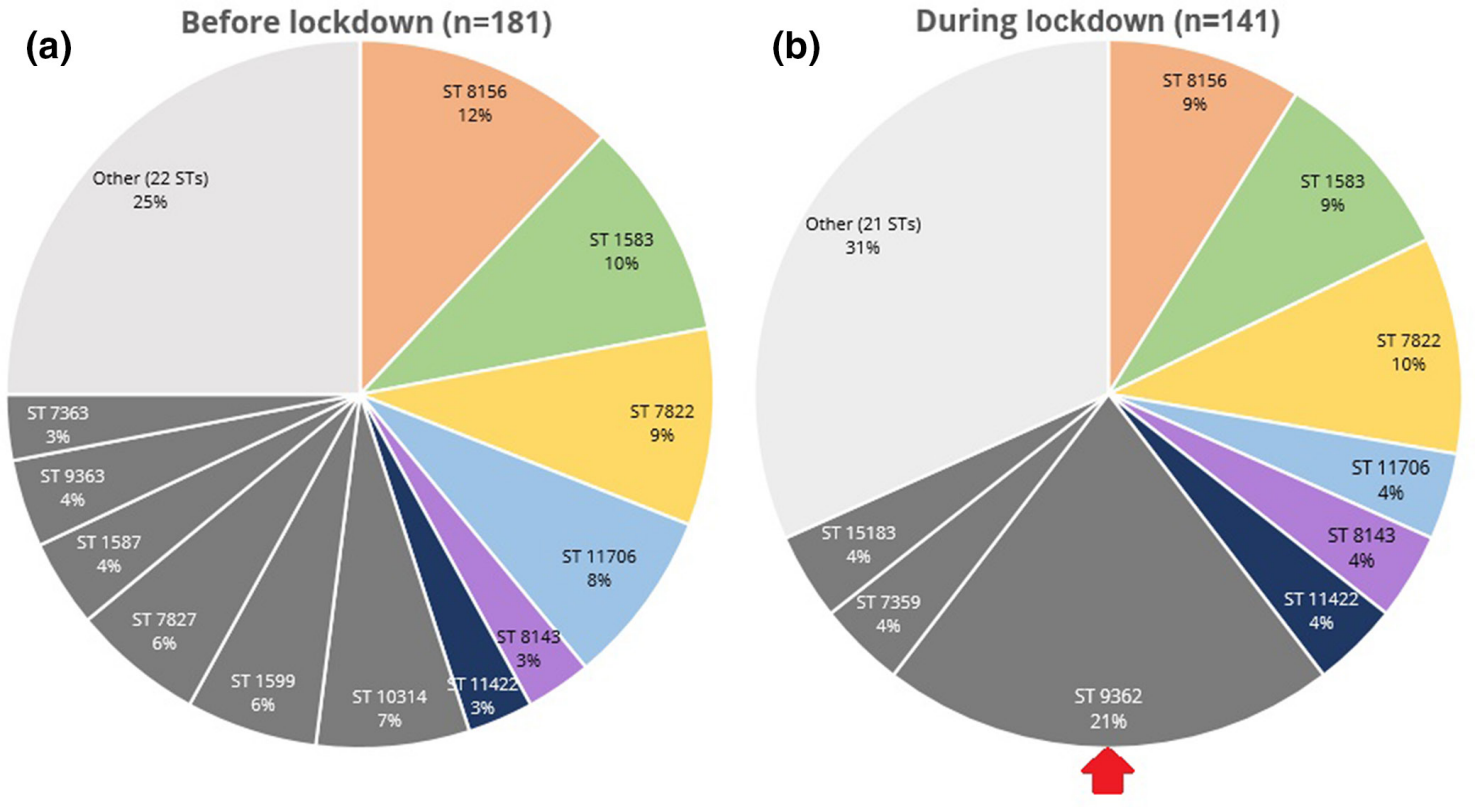
ST distribution before (**a**) and during (**b**) lockdown. Prevalence is shown for STs that were found more than five times in the isolates before or during lockdown. STs that were found fewer than times were categorized as ‘Other’. Coloured STs were found more than five times before and during the lockdown, whereas grey STs are only found more than five times in one of the periods. The arrow indicates ST 9362, which was the predominant ST during lockdown.

All 322 isolates were typed according to the ST scheme. Diversity in STs decreased slightly during the lockdown, from a Simpson’s diversity index of 0.94 before lockdown to 0.92 during lockdown. The number of different STs identified was 34 before lockdown and 30 during lockdown (Fig. 1). The majority of the types were found fewer than five times, which was the case in 22/34 (65 %) STs before lockdown and 21/30 (70 %) during lockdown. Before lockdown, a variety of STs was prevalent among the population, with ST 8156 predominantly found in 22/181 (12 %) of the isolates. During lockdown, ST 8156 prevalence was reduced to 13/141 (9 %), whereas ST 9362 became predominant. ST 9362 went from being present in 3/181 (2 %) of the isolates before the lockdown to 29/141 (21 %) during the lockdown (Fig. 1). ST 9362 was associated significantly with MSM (Table S3). A decline was also found for ST 7827, which went from being present in 10/181 (6 %) isolates before the lockdown to 3/141 (2 %) isolates during the lockdown. The low prevalent STs 1587, 1599, 7363, 9363, 7827 and 10314 were no longer found more than five times during the lockdown, whereas STs 7359, 9362 and 15 183 were found fewer than five times before the lockdown and their prevalence increased during lockdown.

### SNP distances between isolates with identical STs decreased significantly during lockdown

**Fig. 2.**
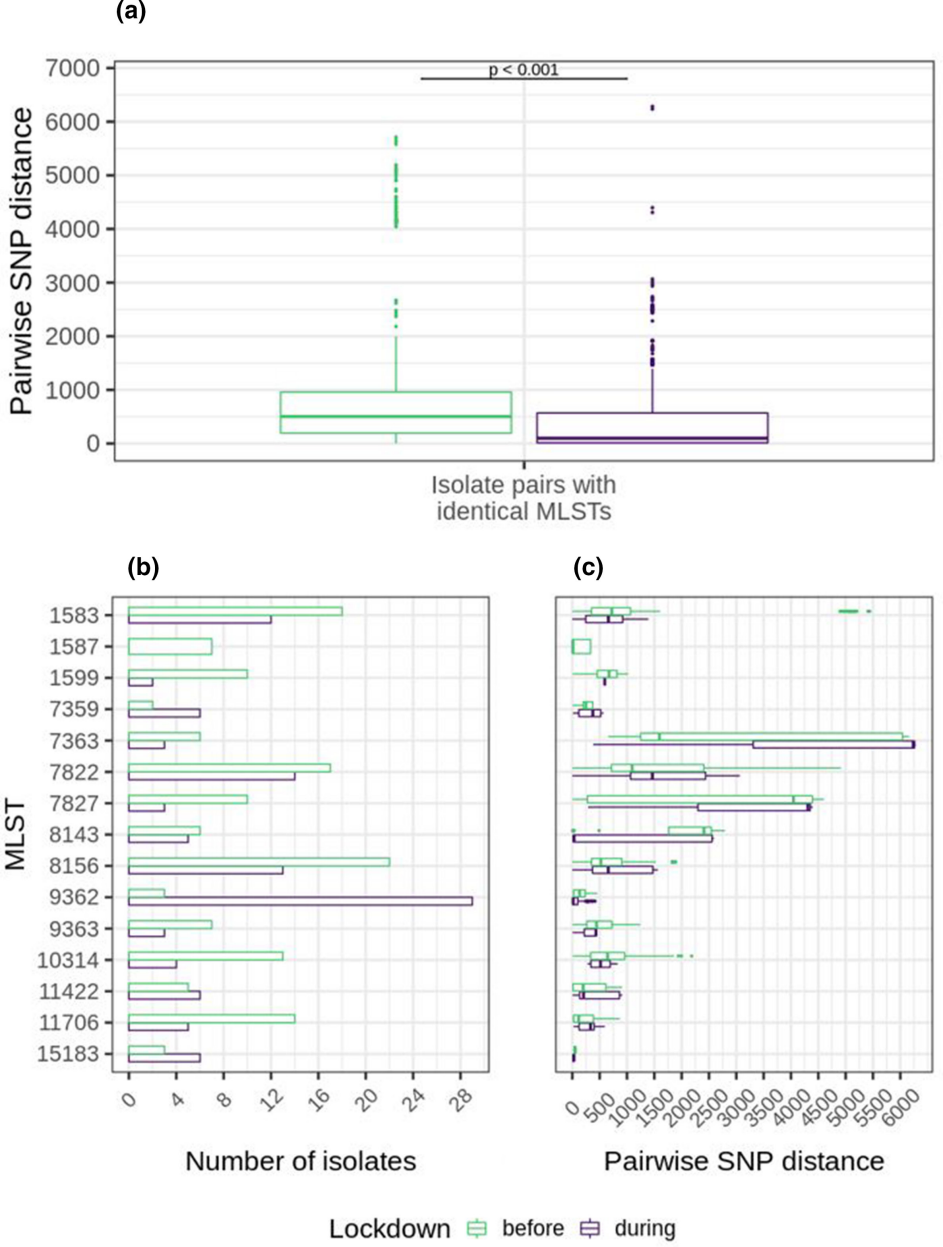
Pairwise SNP distances between isolates obtained before or during lockdown. (a) Boxplots of pairwise SNP distances between isolates with identical STs (901 pairwise comparisons before lockdown, 742 during lockdown). (b) Number of isolates per ST before or during the lockdown. STs found fewer than five times were not shown. (c) Boxplots of pairwise SNP distances between isolates within ST groups before or during lockdown.

**Fig. 3.**
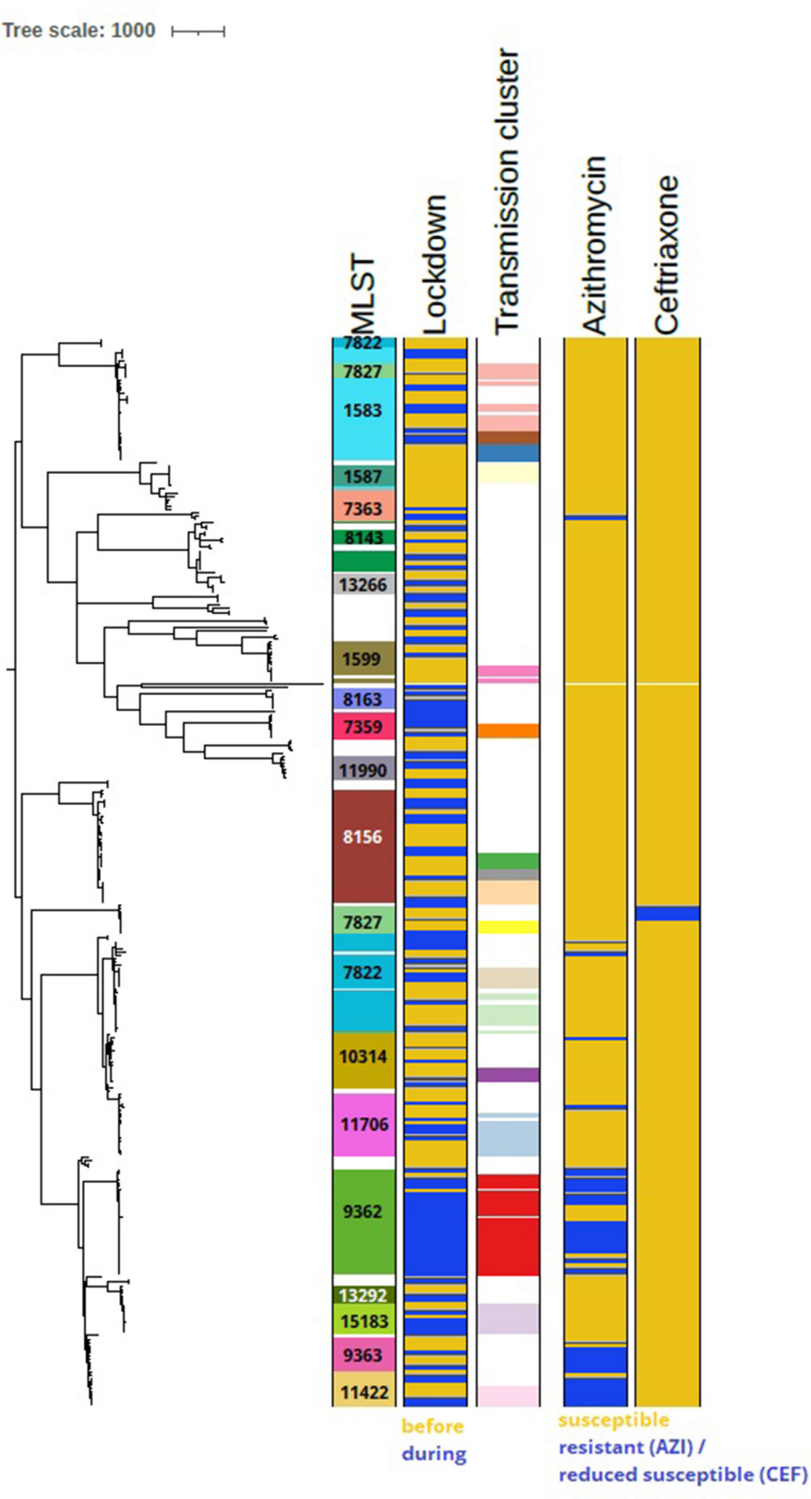
Mid-point rooted core-SNP phylogenetic tree containing all 322 isolates isolated before and during lockdown with corresponding metadata. SNPs were determined by aligning to reference genome FA1090. Transmission clusters were defined as networks of isolates with <10 recombination filtered SNPs between them.

Pairwise SNP distances were determined between isolates obtained before and during lockdown. SNP distances decreased significantly between isolates with identical STs during lockdown, with median SNP distances of 503 before lockdown and 98 during lockdown (Fig. 2a). When further categorizing on ST, a remarkably low median SNP distance of 17 SNPs was found between ST 9362 isolates obtained during the lockdown, despite the high number of ST 9362 isolates found in that period (Fig. 2b, c). Low SNP distances were also found between ST 15183 isolates (median 29 SNPs), but this ST was found only six times during the lockdown. Remarkably, diverse SNP distances were found between isolates with STs 7363, 7822, 7827 and 8143 (Fig. 2c). Using recombination filtered SNP distances, potential transmission clusters were defined which consisted of networks of isolates that differed by <10 filtered SNPs. The percentage of isolates that belonged to a cluster was significantly higher during lockdown (75 %; 106/141) compared to before lockdown (63 %; 114/181) (*P*=0.020, Chi-square test). A total of 53 clusters were identified, of which 17 clusters contained more than three isolates (Fig. 3). The largest cluster contained 28 isolates with ST 9362, of which 96 % (27/28) was obtained during the lockdown.

### Association between genotype and phenotype

**Fig. 4.**
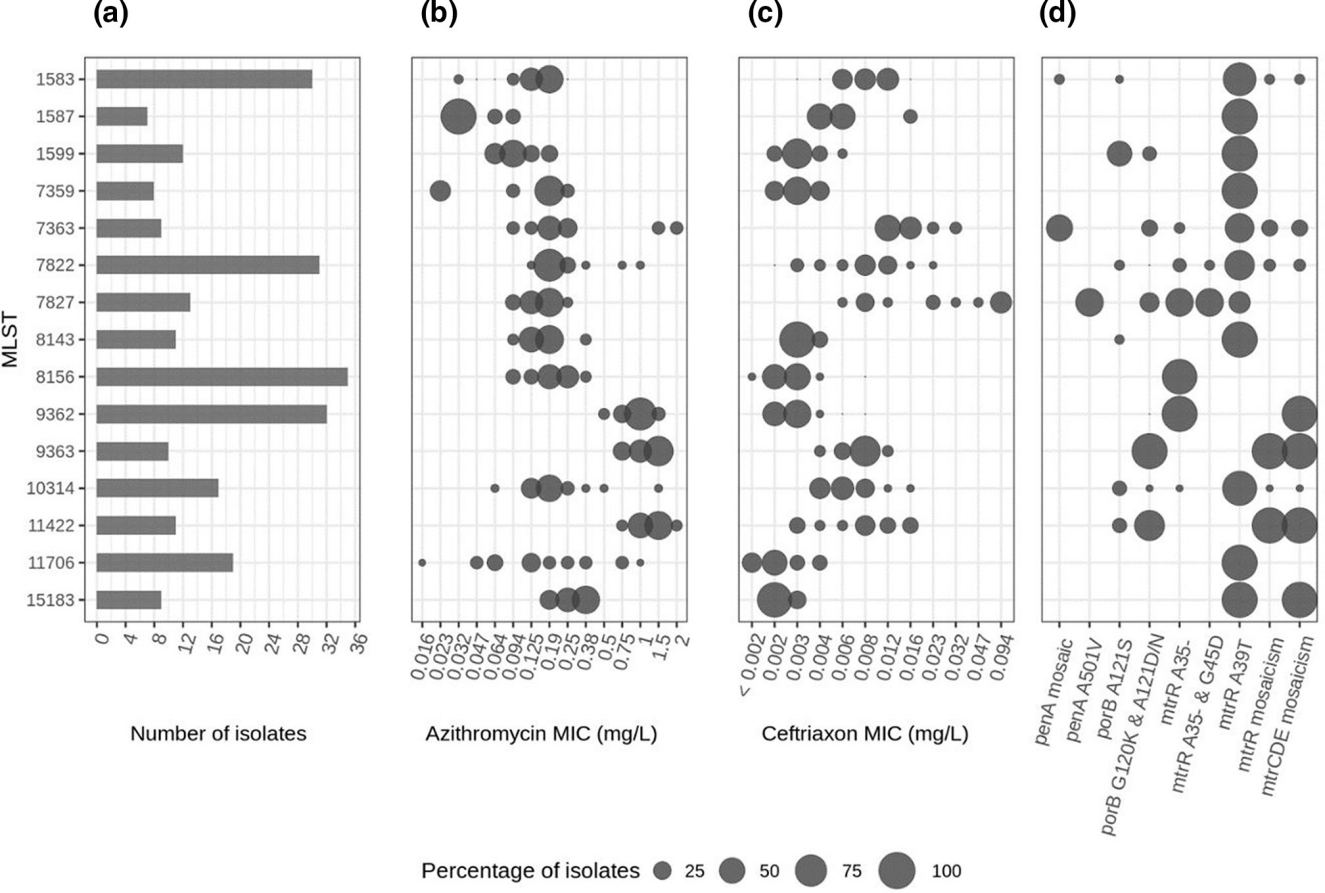
Distribution of phenotypes and resistance determinants among STs. (a) Total number of isolates per ST (numbers before and during lockdown were taken together). (b) Prevalence of azithromycin MICs per ST. (c) Prevalence of ceftriaxone MICs per ST. (d) Prevalence of azithromycin and ceftriaxone resistance determinants in *penA, porB* and *mtrR* genes and the mtrCDE operon per ST. Only STs with more than isolates are shown.

The majority of isolates within an ST had azithromycin and ceftriaxone MICs within a range of 2 dilutions (Fig. 4a, b, c), showing an association between genotype and phenotype. Azithromycin resistance (MIC ≥1 mg l^−1^) was predominantly found in isolates belonging to the STs 9362, 9363 and 11422. Mosaicism in the *mtrR* gene and/or the mtrCDE operon were found to be the resistance determinants in these three STs (Fig. 4d). Azithromycin resistance-associated 23S rRNA mutations were not found. The highest ceftriaxone MICs were found for ST 7827 isolates, which predominantly carried the *penA* A501V mutation. Isolates belonging to STs 8156 and 9362 were highly susceptible to ceftriaxone. No ceftriaxone-resistant isolates were identified in this study.

### Increased azithromycin resistance and ceftriaxone susceptibility during the lockdown

**Fig. 5.**
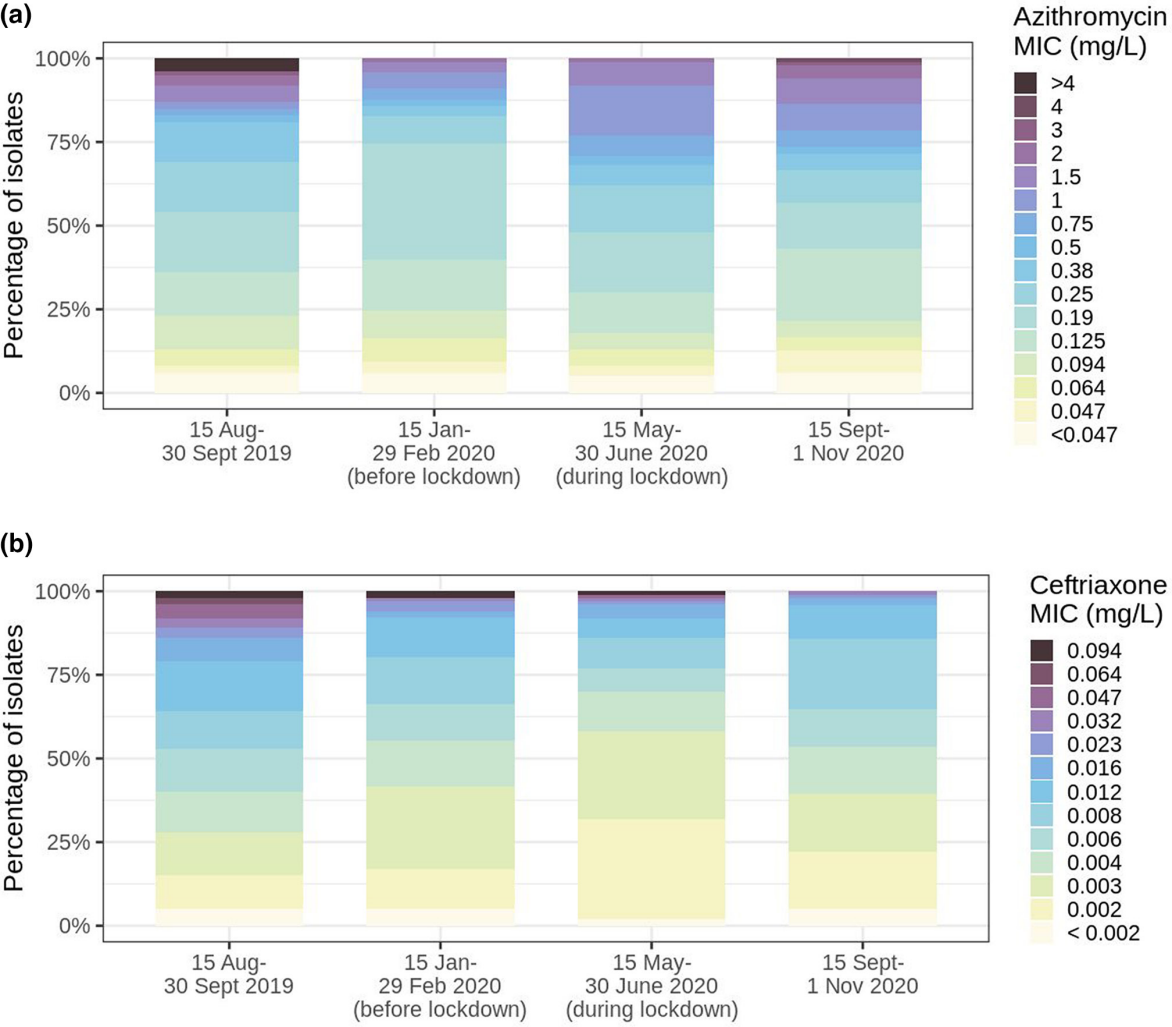
Distribution of azithromycin (**a**) and ceftriaxone (**b**) MICs before, during and after the study periods with intervals of 2.5 months.

The proportion of isolates with low-level azithromycin resistance and ceftriaxone susceptibility increased during the lockdown (Fig. 5a, b). To determine whether this increasing trend was already seen before the study period or was still present in the months afterwards, we compared the MIC distribution of isolates before and during the lockdown with MIC data regarding 163 anogenital isolates cultured 2.5 months before our study period and 167 anogenital isolates cultured 2.5 months after our study period. The azithromycin MIC distribution showed that isolates with high-level azithromycin resistance (MIC >4 mg l^−1^) had already disappeared before the study period (Fig. 5a). However, the proportion of low-level resistant isolates (MIC 1–4 mg l^−1^) increased strongly during the lockdown and the high prevalence remained 2.5 months after the study period. An opposite trend was observed for ceftriaxone MICs, with an increase in ceftriaxone-susceptible isolates and a decrease in intermediate susceptible (MIC 0.012–0.064 mg l^−1^) and reduced susceptible (MIC >0.064 mg l^−1^) isolates over time. Highly susceptible isolates were predominant during lockdown. Reduced susceptible isolates were no longer found after the lockdown (Fig. 5b).

## Discussion

In this study we investigated whether changed sexual behaviour of CSH clients during the first lockdown in Amsterdam, the Netherlands, had led to changes in genotypic and phenotypic distribution within the *Ng* population. A change was observed in ST distribution with the largest shift for ST 9362, which went from a prevalence of 2 % before to 21 % during the lockdown and was exclusively present among MSM. When comparing the ST distribution found in our study to the one found in 2018 in Amsterdam as part of the European Gonococcal Antimicrobial Surveillance Programme (Euro-GASP), the ST distribution before lockdown was much more similar to the one in 2018 than the ST distribution found during the lockdown (Fig. S1) [21]. Most STs that were prevalent in the period before the lockdown were already prevalent in 2018 and no isolates from the Netherlands in the 2018 collection belonged to ST 9362. Therefore, the rapid emergence of ST 9362 does not just reflect a natural trend in ST patterns but may be a result of the COVID-19 lockdown and distance measures. In addition, the limited genetic diversity found among the ST 9362 isolates during the lockdown also suggested extensive local transmission of this strain during the lockdown period. This could be explained by the restricted national and international travel, which logically reduced introduction of *Ng* isolates from sexual networks outside of Amsterdam and the Netherlands. In addition, the significantly reduced number of casual sex partners during the lockdown, as described by Van Bilsen *et al.* [9], may have resulted in a more concentrated sexual network leading to this local transmission. The behavioural changes were only temporary [22], whereas phenotypic data showed that the prevalence of low-level azithromycin resistance remained high after the study period (Fig. 5a), suggesting that ST 9362 remained prevalent. Additional genotypic data are needed to confirm whether easing of measures has led to a decrease of this ST over time or whether this ST is still predominant in Amsterdam.

ST 9362 was associated with low-level azithromycin resistance and ceftriaxone susceptibility and its emergence therefore drove the observed shift in phenotypic data, with increased low-level resistance to azithromycin and increased susceptibility to ceftriaxone during the lockdown. This is further enhanced by the decline in STs associated with azithromycin susceptibility during the lockdown, such as STs 1587, 1599 and 10314 (Figs 2b and 4). The phenotypic shift towards increased low-level azithromycin resistance in *Ng* that has been observed in the Netherlands over the years 2019 and 2020 has also been observed in other European countries and worldwide [21, 23]. European genomic surveillance, which formed part of the Euro-GASP, described that this phenotypic change was caused by the emergence of STs 9363 and 11422, both carrying mosaic *mtrR* and *mtrD* genes, which have been associated with azithromycin resistance [24]. ST 9363 became the predominant MLST in Europe in 2018 [21]. Interestingly, in our study we found ST 9362 being mainly responsible for the increased azithromycin MICs instead, which only carried the mosaic *mtrD* gene and not the *mtrR* mosaic gene. However, we might have missed ST 9363 isolates because our study did not include pharyngeal swabs, and ST 9363 was associated with pharyngeal *Ng* infections in MSM [21]. This could have led to a bias in the MLST distribution that we observed in our study population, but it would only enforce the observed phenotypic shift towards increased azithromycin MICs.

The increased ceftriaxone susceptibility observed in this study was reflected by a decline in ST 7827, which emerged among the Dutch *Ng* population between 2017 and 2019 and was associated with reduced ceftriaxone susceptibility [25]. The trend towards increased ceftriaxone susceptibility observed in this study was also supported by European surveillance data [21].

During the lockdown, more symptomatic patients were seen at the CSH, which may be have been a temporary effect from the prioritization of symptomatic patients during the strict lockdown, due to the reduced capacity for STI testing. To our knowledge, no association has been found between having symptoms and *Ng* genotype, and thus we do not expect a major effect of this change in patient population based on the results of this study. Although we cannot exclude the possibility that the composition of the patient population influenced the *Ng* epidemiology, we tried to minimize this effect by selecting study periods based on the number of isolates that were obtained at the CSH and the similarity of patient populations regarding sex and sexual orientation. Only few isolates were obtained in the first months of the lockdown, due to precautions taken at the CSH, and therefore this period was not chosen. We therefore selected May and June as the period ‘during lockdown’ instead of the first months of the lockdown (second half of March and April), in which the number of *Ng* isolates obtained was strongly reduced and the patient population was much less representative. Since we included isolates from all of the patients who visited the CSH during the study periods, we did not introduce any selection bias in our study.

This study did not include pharyngeal isolates because pharyngeal swabs were no longer taken during lockdown to prevent SARS-CoV-2 transmission. Treatment failures often occurred with pharyngeal *Ng* infections [26]. Pharyngeal *Ng* infections are also important because of the possibility for antimicrobial resistance development due to possible exchange of genetic material between *Neisseria* species at the oropharyngeal site [27]. The Euro-GASP study indeed found an association between pharyngeal isolates from MSM and low-level resistance to azithromycin [21]. The lack of pharyngeal isolates in this study could therefore have led to missed isolates with elevated MICs.

In conclusion, a major change in genotypic and phenotypic distribution was identified during the first COVID-19 lockdown among *Ng* isolates from CSH patients in Amsterdam. The phenotypic shift towards increased azithromycin resistance and increased ceftriaxone susceptibility is in line with European surveillance data, but the lockdown might have enhanced these trends as it has led to expansion of the ST 9362 strain with low-level azithromycin resistance and high ceftriaxone susceptibility. The low SNP distances between ST 9362 isolates suggested local transmission of this strain in Amsterdam, reflecting restricted travel and the previously identified change in sexual behaviour and subsequent more local STI transmission networks during the lockdown [9]. This shows that public health measures also have consequences for the epidemiology of other infectious diseases, which should be taken in consideration for public health surveillance.

## Supplementary Data

Supplementary material 1

Supplementary material 2
