# Supplementary material for: Increased clonality among Neisseria gonorrhoeae isolates during the COVID-19 pandemic in Amsterdam, the Netherlands
Source: Microb Genom. 2023 Apr 6;9(4):mgen000975. doi: 10.1099/mgen.0.000975 (PMC10210945; doi:10.1099/mgen.0.000975)
Supplement: Supplementary material 1 [file mgen-9-975-s001.pdf]

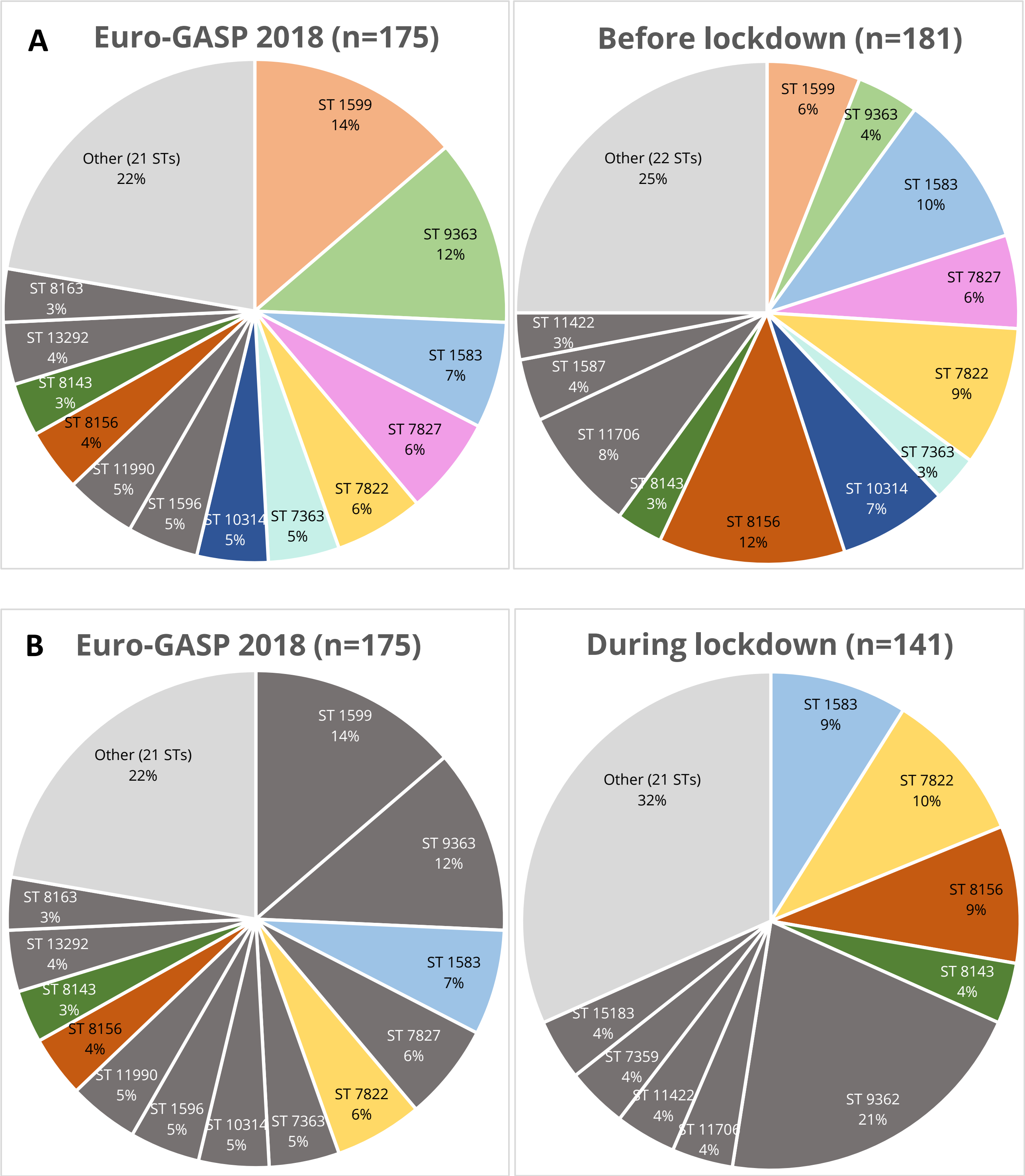

Supplementary figure 1. ST distribution of isolates from Amsterdam in the Euro-GASP 2018 collection compared to the ST distribution of isolates obtained before (A) and during (B) lockdown. Prevalence are shown for STs that occurred >5 times in the isolates before- or during lockdown. STs that occurred <5 times were categorized as ‘Other’. Coloured STs were found > 5 times in both periods that were compared, whereas grey STs are only found >5 times in one of the periods.

Table S2. Timeline of the main COVID-19 events in the Netherlands in the study period of January – July 2020.

Date (DD/MM)* 2020	Measures*
27/02	First notified Sars-CoV-2 infection in the Netherlands
11/03	WHO declares COVID-19 to pandemic
12/03	Events with more than 100 people were cancelled, advice to work from home
15/03	Sport clubs, sex clubs, coffeeshops, bars and restaurants closed
16/03	First lockdown with additional measures: work from home, 1.5 meter distance, schools closed
17/03	Advice to not travel unless urgent, no flights to specific areas "at risk" (continuously updated travel restrictions)
18/03	EU closed borders for non-essential travel, face masks at work
23/03<br><br>*Healthcare CSH Amsterdam*	"Intelligent lockdown" with reminder measures and additional measures: maximum of 3 people visiting a household per day with 1.5m distance, no groups larger than 2 people outside, all events cancelled, all contact jobs closed (for example hairdresser, beauty specialists), 'non-essential' shops, casino's closed<br><br>*No routine HIV/STI testing for asymptomatic clients unless urgent PrEP prescription needed*
09/04	Travelers from "high risk" areas 14 days in quarantine at home
11/05	Elementary school, libraries open and fitness outside allowed with 1.5 meter, contact jobs can start again
01/06<br><br>*Healthcare CSH Amsterdam*	Easing measures: cinema's, theatres, bars and restaurants open with maximum 30 people who reserved a timeslot and keep 1.5m distance, Secondary school opens with distancing measures, obligatory face masks on public transport<br><br>*Open for all clients*
15/06	Easing travel restrictions between EU borders, extension closure of EU outside borders
01/07	Sex workers can start again, sport clubs are opened with 1.5m distance, bars, restaurants and indoor events are allowed to welcome up to 100 people with 1.5m distance, outdoor events up to 250 people with 1.5m distance

CSH: Centre for Sexual Health
* Data from the Dutch government website (https://www.rijksoverheid.nl/onderwerpen/coronavirus-tijdlijn)

Table S3. Statistical association of ST 9362 with epidemiological characteristics of patients from the CSH in Amsterdam before and during the COVID-19 lockdown.

	**Isolates with STs other than ST 9362 (N = 290)**	**ST 9362 isolates (N = 32)**	**P - value**
**Sex**			0.235
Male	272	32	
Female	18	0	
**Age**			0.880
<25	63	7	
25-34	127	14	
35-44	68	9	
>=45	32	2	
**Country of origin**			0.516
The Netherlands	141	17	
Suriname + Dutch Antilles	35	1	
Europe + Turkey	51	5	
M-S America	28	3	
Asia	19	5	
Africa	11	1	
Other	4	0	
Unknown	1	0	
**Sexual orientation**			0.021*
MSM + Transgender	250	32	
MSW + Female	40	0	
**HIV status**			0.503
Negative	214	27	
Positive	55	4	
Unknown	21	1	
**Sex work**			0.492
Yes	25	1	
No	265	31	
**Number of sex partners in last six months**			0.592
0-1	14	2	
2-4	80	5	
5-9	72	11	
10-19	49	7	
20-49	55	5	
>=50	20	2	
**Symptomaticity**			0.442
Symptomatic	97	8	
Asymptomatic	193	24	

* $p \leq 0.05$
